# Experimental and Simulation Study on Welding Characteristics and Parameters of Gas Metal Arc Welding for Q345qD Thick-Plate Steel

**DOI:** 10.3390/ma16175944

**Published:** 2023-08-30

**Authors:** Hui Zhang, Rong Li, Shuxuan Yang, Liebang Zhan, Ming Xiong, Ban Wang, Juyong Zhang

**Affiliations:** 1 School of Mechanical Engineering, Hangzhou Dianzi University, Hangzhou 310018, China; zhhui612@163.com (H.Z.); mycaps_2013@163.com (S.Y.); zlb517516@163.com (L.Z.); banwang1212@sina.com (B.W.); zhang_juyong01@163.com (J.Z.); 2 CAS Key Laboratory of Solar Activity, National Astronomical Observatories, Beijing 100012, China; 3 State Key Laboratory of Space Weather, National Space Science Center, Chinese Academy of Sciences, Beijing 100190, China; mxiong@swl.ac.cn

**Keywords:** thick-plate bridge steel, GMAW, welding process parameters, thermal–mechanical coupling

## Abstract

The welding and construction processes for H-type thick-plate bridge steel involve complex multi-pass welding processes, which make it difficult to ensure its welding performance. Accordingly, it is crucial to explore the inherent correlations between the welding process parameters and welding quality, and apply them to welding robots, eliminating the instability in manual welding. In order to improve welding quality, the GMAW (gas metal arc welding) welding process parameters are simulated, using the Q345qD bridge steel flat joint model. Four welds with X-shaped grooves are designed to optimize the parameters of the welding current, welding voltage, and welding speed. The optimal welding process parameters are investigated through thermal–elastic–plastic simulation analysis and experimental verification. The results indicate that, when the welding current is set to 230 A, the welding voltage to 32 V, and the welding speed to 0.003 m/s, the maximum deformation of the welded plate is 0.52 mm, with a maximum welding residual stress of 345 MPa. Both the simulation results of multi-pass welding, and the experimental tests meet the welding requirements, as they show no excessive stress or strain. These parameters can be applied to building large steel-frame bridges using welding robots, improving the quality of welded joints.

## 1. Introduction

Welding plays an important role in manufacturing and industry, especially with the advent of intelligent welding technology. Welding robots enable real-time monitoring and control, to improve productivity and weld quality. With the help of cloud computing and big data, remote control and intelligent management can also be achieved. Therefore, an in-depth study of the relationship between welding factors is needed, to improve welding performance and achieve high-performance manufacturing.

The welding quality of large thick-plate Q345qD bridge steel, typically used in H-type structures, is significantly affected by the GMAW (gas metal arc welding) welding process parameters. These parameters play a crucial role in ensuring the safety and durability of bridges. Therefore, when employing welding robots in bridge construction, it is essential to have a clear understanding of the relationship between the welding process parameters and the resulting welding quality.

The H-type steel structure has the advantage of comprising simple components, which saves labor and materials, and has become an increasingly widely used steel structure type in modern new economic bridge structures, industrial buildings, and power station construction. The production and construction of bridge steel mainly adopt the welding connection mode. Therefore, the performance of the welded joint directly affects the safety and lifespan of the entire steel component building [1,2]. The thick steel plate uses the multi-pass welding process and GMAW, in which the welding process parameters are the main factors affecting the welding temperature field and stress–strain field, mainly including the welding current, welding voltage, and welding speed. The welding process parameters relate to the parameter setting of the welding current, welding voltage, and welding speed, to avoid uneven heating or excessive residual stress and strain concentration in the welding geometric model. There are many methods to optimize the welding parameters, including theoretical analysis, simulation, and testing. Bibin, [3] adopted a novel dual-pulse gas metal arc welding (DP-GMAW) technique, which reduces the heat input during welding, using the current pulsing approach. The dual-pulse approach was capable of a 67% reduction in heat input, and a 49% reduction in RA, compared to conventional gas metal arc welding (GMAW). Farzaneh S’s experimental test was to assess the residual stress by changing the direction, along with multiple welding passes, and determine the fatigue life of the welded joints. The result shows that compressive residual stress increases in the sample, gradually, from the single-pass weld to the double- and triple-pass weld [4]. Aditya, K., [5] evaluated of the influence of the welding current on tungsten inert gas (TIG) welding and activated tungsten inert gas (ATIG) welding, and studied on the depth of penetration and microstructure properties were performed. Various super-duplex stainless steel (SDSS) beads on plate weld joints were studied. Visual observation showed that a lower welding current resulted in partial weld penetration in the case of both the TIG and ATIG weldments. The weld joints obtained via the ATIG process have a higher depth of penetration and depth/width ratios than the weld joints obtained via the conventional TIG process at comparatively lower welding currents. Thien, N.T., [6] describes a welding method that is preferable to automatic rotary welding as an alternative to the traditional manual welding method. This greatly improves the quality of the weld, and reduces the welding time. Deepak. K., [7] compares the penetration, reinforcement, and weld-bead width of TMHW with metal active gas welding (MAGW). The results show that TMHW yields a higher penetration and weld-bead width with less reinforcement height than MAGW. Merbin, J.’s, [8] article discusses fusion-based welding techniques, such as gas tungsten arc welding (GTAW) and laser beam welding (LBW), and solid-state welding techniques, such as friction stir welding (FSW) and explosive welding (EB), for a broad category of HEAs. In addition, the microstructural features and mechanical properties of HEAs welded using different techniques were explained for a broad spectrum of HEAs. Subhash, D., [9] introduced the Regulated Metal Deposition (RMD™) process, a variant of the gas metal arc welding (GMAW) process developed to effectively control metal transfer in short-circuit mode. The process is essentially a modified short-circuit GMAW process with uniform droplet deposition, making it easier for the welder to control the molten pool, and thereby improving the quality of the welded joint. Maksim, L., [10] studies the laser welding of thin-thickness Ti-6Al-4V parts, manufactured via selective laser melting (SLM). A full factorial experiment was carried out in order to construct a regression model of the technological parameters (the laser power, welding speed, and defocusing amount) which influence the weld shape. A metallographic analysis was carried out, and it was found that the thermal cycles of product printing and laser welding were equivalent. The microhardness analysis also showed no differences between the weld metal and the base metal. R.S.B., [11] have made an attempt to identify the requirements of submerged arc welding (SAW), its arc–column behaviors, its varied effect on the weld quality, the characterization of defects using sensor signals, the methods used for monitoring, and techniques to construct an intelligent welding expert system using Internet of Things (IoTs). Aravind, [12] conducted experimental research on cold-metal transfer (CMT) welding on Al5083 thin plate. With the adoption of the L9Taguchi orthogonal test design, the welding current (A), welding speed (mm/min), and welding frequency (Hz) are taken as input parameters for CMT welding. The weld quality is studied via measuring the reinforcement, weld width (BW), penetration depth (DOP), and heat-affected zone width (HAZW). The optimized parameters are found via the VIKOR multi-objective optimization method. Frango, L.T., [13] has proposed metal inert gas (MIG) welding as an advanced welding method to weld quality joints with good strength. Moreover, it is easy to weld hard materials using this technique. The current, voltage, and speed are the basic factors that affect or change the strength of the weld. The objective of this paper is to maximize the welding strength using optimum input parameters. In Tarık H S’s study [14], the weldability of 10 mm thick 316 L stainless steel plates used in the production of LNG tanks via gas tungsten arc welding was investigated. The effect of multi-pass welding on the microstructural evolution and, thus, on the mechanical behavior of the joint produced, was studied. Optical microscopy and scanning electron microscopy, as well as energy dispersive spectroscopy, were employed to investigate the microstructural evolution in the weld region of the fabricated joint. Kanakavalli, B.P., [15] presented the application of the Taguchi and grey relational analysis methods in determining the optimum process parameters for MIG welding. The Taguchi method is widely used for designing optimum experiments, while grey relational analysis is used for decision making in cases of considering multiple criteria, and the combination of these two methods becomes an effective tool for determining the optimum process parameters. Zhu, [16] optimized the process parameters using hybrid fiber laser–MIG welding for A7N01 plates with the dimensions of 500 × 450 × 8 mm after welding. The plates are in a V groove, with a 1 mm blunt edge. Rizvi, [17] focused on optimizing different welding parameters through Taguchi technology, getting an optimum technological parameter of 23 V, wire stroke of 350 IPM, and gas flow of 15 L/min. At present, there is limited research on welding quality control in thick-plate bridge steel, due to its large structure size, its multi-pass welding, its complex and diverse process parameters, its difficult processing, the complex and diverse metal chemical elements in the weld structure, and the uneven thermal characteristics of the materials when simulated by numerical simulation.

Therefore, this study focuses on Q345qD, a large-scale thick-plate bridge steel, and utilizes ABAQUS-2022 simulation software, the thermal–elastic–plastic finite element method, and FORTRAN-95, to develop a welding simulation program. The objective is to optimize and simulate the GMAW process parameters for bridge steel, identify the optimal welding process parameters, and investigate their effects. Through optimizing the welding process parameters, the study aims to enhance the quality of the welded joints, and prevent welding defects.

## 2. Material and Research Method 

### 2.1. Definition of Welding Process Parameters

In the welding process of developing multi-pass welds to large thick plates, welding process parameters are one of the main factors affecting the microstructure and properties of the weld joints. Using Q345qD steel, we studied the influence of GMAW welding process parameters via the thermal–elastic–plastic finite element method. The welding geometric model size is 300 × 250 × 20 mm, and the groove shape is an “X” with an angle of 60°. According to the standards of the Code for Design of Steel Structures of Railway Bridges (TB10091-2017) and Symbol Representation of Welds (CB/T324-2008), the welds are set to be four layers and four passes, which are welded via gas-shielded welding and submerged-arc welding. In actual welding, because of the combination of weld and base metal, it is difficult to define the contour of the weld in simulation, so the simulation model is simplified, to keep the welding sequence and welding boundary conditions unchanged during the loading process of the welding heat source.

### 2.2. Methodology

**Figure 1:**
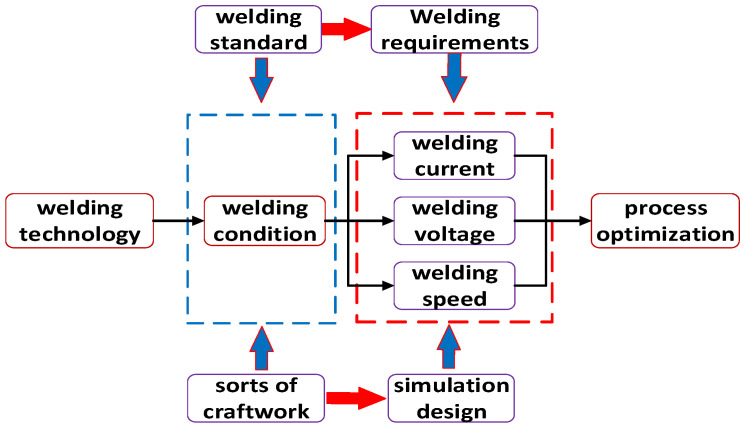
Flow chart of the welding process parameter research method.

Regarding the GMAW welding method of Q345qD thick-plate bridge steel, a scheme design and numerical simulation analysis of the welding process parameters are carried out. In the actual welding process, the parameters include the current, voltage, and speed, which vary depending on the welding methods and objects. During the welding simulation, the welding process parameters play a crucial role in determining the welding heat and weld formation. Therefore, it is essential to analyze and optimize the welding process parameters for the design, as this significantly impacts the welding quality. The optimal values are selected on the basis of the simulation results for each parameter, and the preferred solution is finally built. Due to the complexity of the welding condition factors, instead of all the welding parameters being optimized together, each parameter is simulated individually, and the optimum value is selected. The welding current, welding voltage, and welding speed are the three most important parameters, and are also specifically modelled in the following section. The study and process flow are shown in Figure 1.

Combining the optimization scheme of the welding process parameter design with research into simulation thermal–elastic–plastic theory, we adopt ABAQUS software to carry out the optimization simulation operation of the welding process parameters. Because the strain field and stress field are generated through the thermal–mechanical coupling between the temperature field and the material itself, only the influence law of each welding process parameter on the welding temperature field is analyzed here. A complete thermal–mechanical coupling of the temperature field with the different welding process parameters of a single weld is carried out, and the welding process is accurately simulated. The influence of the welding process parameters on the welding process is explored, and the optimal welding process parameter scheme is obtained.

### 2.3. Specification of Parameters

This simulation experiment discusses the simulation of the multi-pass welding of large thick plates. The model size is 300 × 250 × 20 mm, adopting an X-shaped groove, and the thick-plate pass is divided into four welds, which are aligned forward and backward. CO_2_-gas-shielded welding is used in backing the submerged arc welding (SAW). The temperature range of the welding pass is 135~165 °C, the allowable deformation error is 1~2 mm, and the cooling speed is 35~60 °C/s. Referring to the welding Code for Steel Structures (GB/T50661-2011) and Welding Wire of Carbon Steel, Low Alloy Steel for Gas Shielded Electric Welding (GB/T8110-2008), and considering the experienced values in the actual factories, the welding current, welding voltage, and welding speed are proposed, as shown in Table 1.

## 3. Numerical Analysis

### 3.1. Thermal–Mechanical Coupling

The welding process is a coupling way of heat transfer, electromagnetism, and thermodynamics to connect metals or other thermoplastic materials. It is complex and changeable, and contains complex thermodynamic problems. The thermodynamic problems directly affect the composition and distribution of the welding microstructure. According to the classical thermodynamic formula, the thermodynamic heat transfer in the welding process can be divided into three forms: heat conduction, heat convection, and heat radiation [18]. In thermal–elastoplastic analysis, assumptions are made, as follows.

1. The material yield follows the von Mises yield criterion.
2. The elastic strain and plastic strain of the materials can be separated from the temperature strain.
3. The mechanical properties of the materials depend on temperature changes.
4. The behavior of the material in the plastic zone after yielding follows the plastic-flow criterion and strengthening criterion.
5. Based on the small-time increment, the mechanical properties, stress, and strain of materials show linear changes.

In the welding process, with the continuous movement of the welding heat source, the welding material and base metal will fuse with each other, then generate heat during phase transformation. The temperature at each point in the welding process can be expressed as Equation (1).

(1) T=T(x, y, z, t)

where *T* refers to the instantaneous temperature at any point in the welding process (*k*), (x, y, z, t) is the coordinate of a certain point, and *t* is the time (s).

Therefore, regarding the nonlinear transient heat conduction problem, its temperature control equation can be expressed as follows.

(2) $$\rho c\frac{\partial T}{\partial t}=Q+\frac{\partial }{\partial x}\left(k_{x}\frac{\partial T}{\partial x}\right)+\frac{\partial }{\partial y}\left(k_{y}\frac{\partial T}{\partial y}\right)+\frac{\partial }{\partial z}\left(k_{z}\frac{\partial T}{\partial z}\right)$$

where Q(x, y, z, t) is the heat flow in the object is expressed, ρ expresses the welding material density, *c* is the welding material heat capacity, $k_{x}$, $k_{y}$, $k_{z}$ represent the heat-transfer coefficient of the welding materials in three-dimensional coordinates, and *T* is expressed as the differential equation of the transient welding temperature field. 

### 3.2. Welding Heat Source

The heat source model is a characteristic equation for simulating the heat distribution of the welding heat input in the weld direction, which represents the range and form of the heat distribution. The heat source model has a great influence on the simulation accuracy of the welding temperature field and stress–strain field near the heat source [19].

The development of welding heat sources has changed from plane space to three-dimensional space; at this point, heat sources and surface heat sources are often used in plane simulation operations. At present, the bulk heat source model is mostly used for the welding simulation of thick plates, considering the influence of the weld penetration, weld width, heat transfer distribution, and electric strength. 

**Figure 2:**
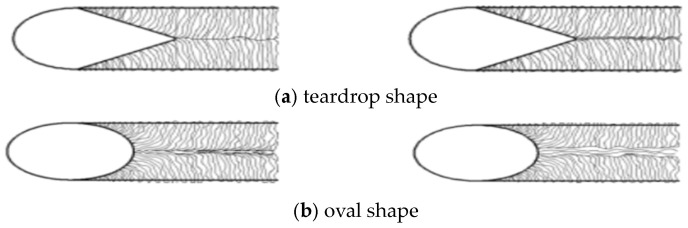
Schematic of Heat Flow.

In the actual welding process, the welded joint organization will change with the change in the welding heat flow, especially the welded joint grain formation, which will first follow the direction of the heat run expansion, or follow the perpendicular to the fusion line isothermal direction of expansion. Temperature line perpendicular to the direction of expansion. Grain molding evolves into a number of different weld-pool shapes, the most common of which are the teardrop and oval shapes, as shown in Figure 2, below. 

Among them, the teardrop-shaped weld pool will form a clear center line; the solidification interface in the center line is prone to cracking ripples, so, in the welding process to avoid the formation of a teardrop shape in the weld pool. Considering the influence of the shape of the weld pool and the advantages of the body heat source comparison, we attempt to make the welding simulation process closer to the welding experimental process, to achieve accurate modeling of the welding process. The simulation of the temperature field and stress–strain field changes in the welding process to avoid the generation of a teardrop-shaped welded molten pool.

Through a comprehensive consideration of the choice of the double-ellipsoid heat source model as a welding simulation heat source model, the shape of the center of the heat source is divided into double-ellipsoidal and spherical, indicating the distribution range of the heat source. The heat density q spreads normally inside the ellipsoid, and the maximum value is located in the ellipsoid, and decreases from inside to outside, in the form of Gaussian arcs.

**Figure 3:**
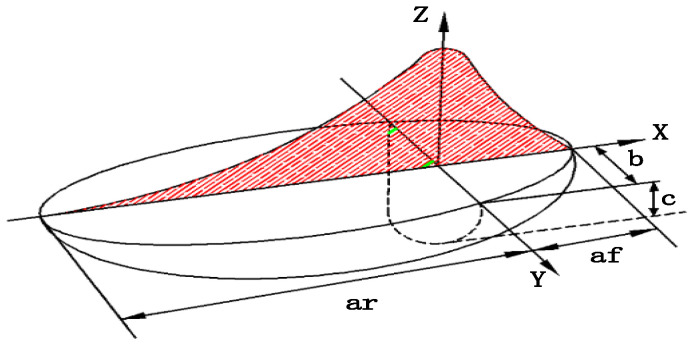
Double-ellipsoid heat source model.

The double-ellipsoid heat source model not only accurately simulates the temperature range in the first half of the actual arc-loading process, which is higher than in the second half, but also accurately simulates the penetration effect of the arc on the weld, which satisfies the accurate simulation of the GMAW welding pool, as shown in Figure 3. The green shadows are the highest points of heat density, and the red color is the tendency for heat density to disperse.

Heat density diffusion function of the first-quarter ellipsoid:

(3) $$q_{f}\left(x,\;y,z\right)=\frac{6\sqrt{3}(f_{f}Q)}{a_{f}bc\pi \sqrt{\pi }}\exp\left(-\frac{3x^{2}}{a_{f}^{2}}-\frac{3y^{2}}{b^{2}}-\frac{3z^{2}}{c^{2}}\right),\;\;\;\;\;x\geq 0$$

Heat density diffusion function of the tail-quarter ellipsoid:

(4) $$q_{r}\left(x,\;y,\;z\right)=\frac{6\sqrt{3}(f_{r}Q)}{a_{r}bc\pi \sqrt{\pi }}\exp\left(-\frac{3x^{2}}{a_{r}^{2}}-\frac{3y^{2}}{b^{2}}-\frac{3z^{2}}{c^{2}}\right),\;\;\;\;\;x<0$$

where Q=ηUI-represents the welding heat input, and η is the welding heat source efficiency. $f_{f}$, $f_{r}$, respectively, represent different proportional functions of the heat of the front and rear ellipsoids, and $f_{f}+f_{r}=2$. $a_{f}$, $a_{r}$ represent the inner diameter parameter of the double ellipsoid. b represents the melting width dimension of the double ellipsoid. c represents the penetration size of the double ellipsoid. $a_{f}$, $a_{r}$, *b*, and *c* represent the shape and size parameters of the ellipsoids, respectively, and are independently selected according to the different weld sizes and welding process parameters, which fully consider the difference in the heat flow energy distribution in the heat source models. 

Where $a_{f}$, $a_{r}$, *b*, and *c* need to be combined with the actual welding model and welding process to set independent values, so *b*, *c* can be set according to the weld size parameters, while $a_{f}$, and $a_{r}$ can be set according to the empirical Formulas (5) and (6), to determine:

(5) $$a_{f}=0.6\times b$$

(6) $$a_{r}=2.0\sim 2.5\times b$$

As it is not possible to directly measure the actual effective range of the arc, the macroscopic dimensions of the weld cross-section are used as the shape parameters of the model, b is the actual melt pool half-width, and *c* is the actual melt depth. The weld is made using an X-bevel, and the weld is four passes. The second weld in the cross of the “X”, so set the melt width for the minimum, the first weld in the second weld on the bias, so the melt width is slightly larger than the first weld, the same reason the third weld, the fourth weld at the outermost end, take the value of the melt width of the largest. The specific parameters selected are set on the basis of actual welding experience. In summary, combined with the welding model size, weld size, and actual welding process, the empirical Equations (5) and (6) are used to set the weld size of the double-ellipsoid heat source model. Therefore, the parameters of the double-ellipsoid heat source model for the four welds are set as shown in Table 2, below.

### 3.3. Secondary Development of Heat Source Model Subroutine

Using the FORTRAN language for the secondary development of the welding subroutine in ABAQUS, which simulates the loading process of the welding heat source, the size of the welding heat source, weld penetration, weld width, and loading direction of the welding heat source are all set in the subroutine. The secondary development of the heat source SUBROUTINE adopts the subroutine in the FORTRAN language, where the subroutine starts at “subroutine”, and uses the end statement. According to four welds, different welding process parameters are set in the subprogram, and the relationship between the heat flow, TIME, and position is formed using the total time control method.

### 3.4. Numerical Modelling

We adopt Q345qD bridge steel, which is a low-alloy, high-strength steel with a carbon content of 0.16%. It has a high strength and toughness, meaning that it can bear the load and impact of rolling stock, with a good fatigue resistance, low-temperature toughness, and atmospheric corrosion resistance. It is a steel specially used in viaducts or highway bridges. Its chemical composition is shown in Table 3.

**Figure 4:**
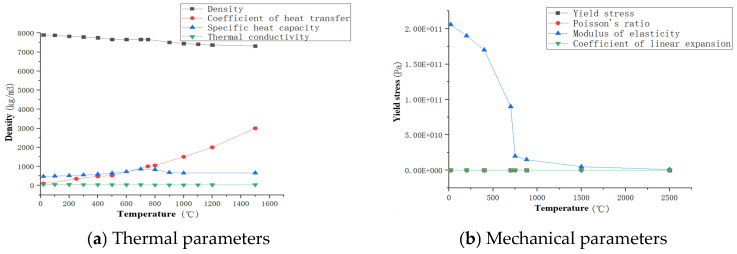
The thermo-physical parameters of the welding parent metal.

The main chemical composition of Q345qD steel is manganese and silicon, with an impact absorption energy of 187 KV^2^/J, yield strength of 397 MPa, and tensile strength of 533 MPa. The welding temperature field and stress–strain field are simulated via the method of butt welding, because the thermal physical parameters of Q345qD bridge steel are distributed differently under different high-temperature conditions. The absolute zero of the numerical simulation is −273.1 °C, the Stephen–Boltzmann constant is 5.67 × 10^−8^, the latent heat of the material is 256,400 J/kg, the radiation heat transfer coefficient is 0.85, and the ambient temperature is 20 °C. Other characteristic parameters of the material, such as the density, specific heat capacity, thermal conductivity, Poisson’s ratio, yield stress, elastic modulus, and linear expansion coefficient are shown in Figure 4.

**Figure 5:**
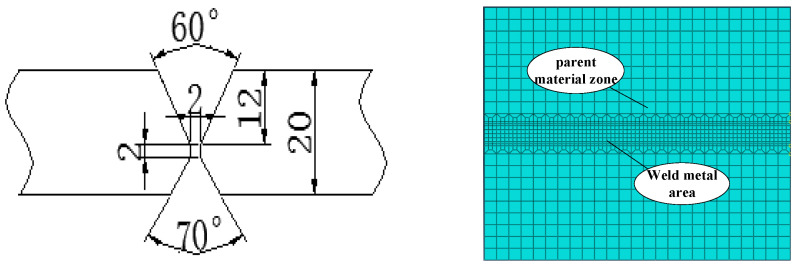
Schematic diagram of the welding model and groove size.

Considering the size of the steel, and the technological characteristics caused by multi-pass welding, we use ABAQUS to establish a flat-butt multi-pass welding model and conduct simulation research, assuming the model is an ideal elastic–plastic rigid body, the material is Q345qD steel, the size is 300 × 250 × 20 mm, and the weld seam and base metal are established by zones. An X-shaped groove is adopted for the groove, and its size and weld-bead number are shown in Figure 5.

### 3.5. Boundary Conditions

**Figure 6:**
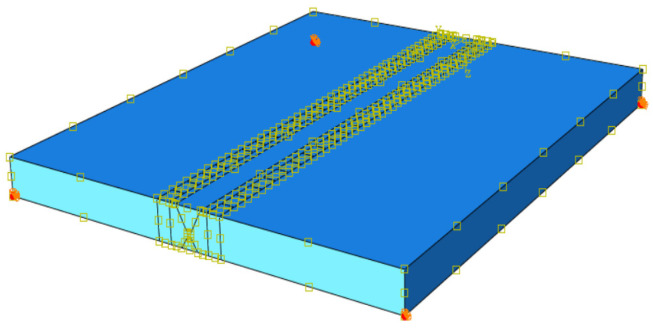
Schematic diagram for the three-dimensional constraint. (The blue plate is the steel, whose four corners at bottom are fixed, and the yellow “X” section is the weld seam.).

Based on the actual welding process, heat conduction is the main method inside the welding material, while convection heat transfer and radiation heat transfer are the main methods outside. Therefore, the environmental temperature is set at 20 °C, which accurately simulates the environmental boundary conditions of the welding model. We restrict its motion boundary and consider its gravitational influence; therefore, the degree of freedom is constrained in three directions to the four vertices in the bottom surface for the multi-pass welding of flat plates, as shown in Figure 6. The yellow part is the actual welding route.

## 4. Simulation Results

### 4.1. Effect of Welding Current

**Figure 7:**
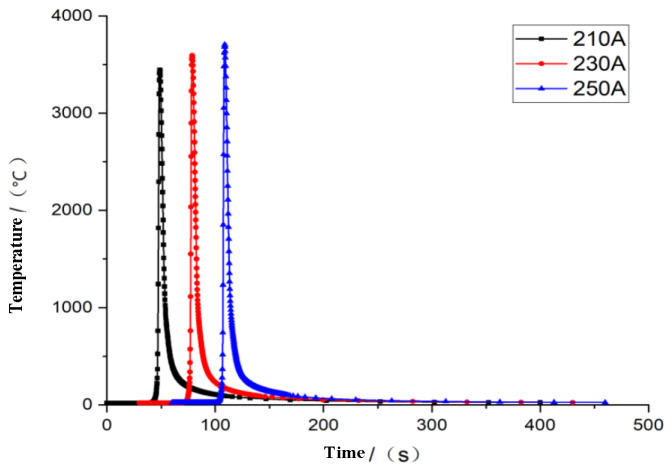
Thermal variation under different currents.

With parameters and welding boundary conditions remain unchanged, during the welding heat source charging process, the welding voltage is set to 32 V, and the welding speed is set to 0.003 m/s, then the current value is taken separately to determine the influence of the welding current on the welding performance. According to the welding method and welding manual specifications, the welding current ranges from 210 A to 250 A. As the boundary value and the center value have a representative influence, the welding simulation is carried out with 210 A, 230 A, and 250 A currents, and the temperature field changes are shown in Figure 7.

As we can see from Figure 7, with the increase in the welding current, there is a greater welding heat; the maximum temperature can reach 3706 °C.

### 4.2. Effect of Welding Voltage

**Figure 8:**
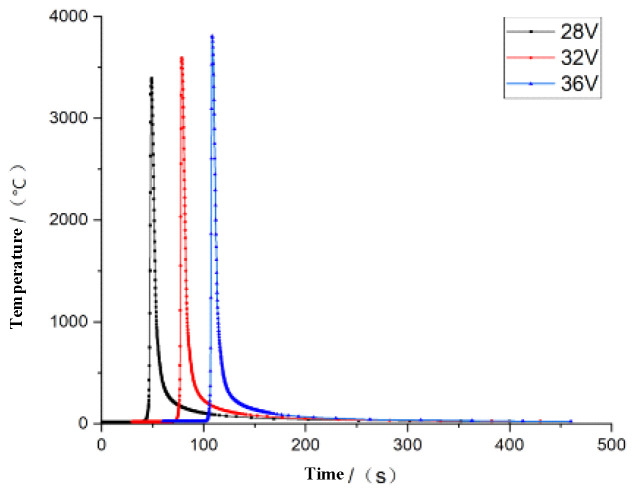
Thermal variation under different voltages.

Similarly, on the premise of keeping the other welding conditions unchanged, if the welding current is 230 A, and the welding speed is 0.003 m/s, then the welding voltage is taken separately to detect the influence of the welding voltage on the welding performance. According to the welding method and welding manual specifications, the welding voltage ranges from 28 V to 36 V. In the same way, a welding simulation is carried out with 28 V, 32 V, and 36 V voltages, and the temperature field changes are shown in Figure 8.

We can see from Figure 8, with the increase in the welding voltage, there is a greater welding heat; the unit node temperature can reach 3807 °C.

### 4.3. Effect of Welding Speed

**Figure 9:**
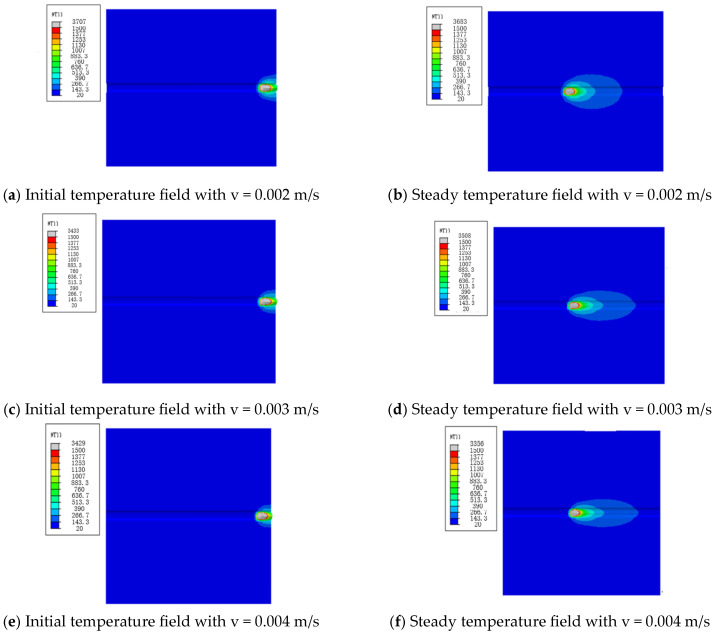
Temperature field distribution at different welding speeds.

In practical welding, the welding speed is different, and it is also a key parameter that affects the welding joints. We select three welding speeds, v = 0.002 m/s, v = 0.003 m/s and v = 0.004 m/s, to investigate the effect of the welding speed, and obtain the results shown in Figure 9.

According to Figure 9, the diffusion shape of the temperature field in the welding results is similar to the elliptical shape, which has nothing to do with the different welding speeds; instead, the area of the elliptical shape is different due to the different loading and running speeds of the welding heat source, which decrease as the welding speed increases. However, with the continuous running of the heat source, the rear part of the temperature field is gradually shortened and reduced, so, if the size of the welding geometry model in the loading direction of the heat source is long enough, the temperature field will gradually appear oval. This is because, with the increase in the welding speed, the welding heat input is small, which causes the maximum temperature of the welded joint to decrease with the increase in the welding speed.

## 5. Optimization Analysis and Experiment

The multi-pass welding simulation is based on the single-pass welding simulation. Combined with the influence of the welding process standards on the welding process parameters and processes, the optimization scheme for the multi-pass welding process parameters is designed as shown in Table 4.

According to Table 4, the numerical simulation of welding is carried out, and the distribution of the temperature field, stress field, and strain field in the simulation is analyzed.

### 5.1. Results of Temperature Field 

**Figure 10:**
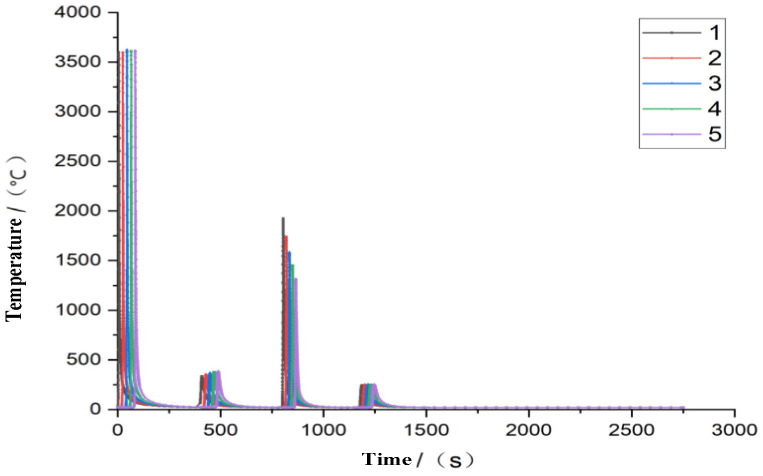
Temperature variation curve of the first weld joint.

In multi-pass welding, there are complex nonlinear changes in the welding process. According to the optimized parameters, five grid nodes are selected in the first welding seam, investigating the thermal variation in the welding, as shown in Figure 10. 

According to the change in the joint temperature, the maximum temperature of the first weld is 3658 °C. With the operation of the heat source, the temperature is gradually transferred to the next weld, while the second weld is on the back of the flat plate, and the temperature is relatively low at 300 °C. When the heat source is operated to the third weld, submerged arc welding is used, the temperature is relatively high, and the distance from the joint is different; the minimum temperature of the joint is 1300 °C. It can be concluded from the welding temperature field that the welding temperature field changes sharply, the cooling speed of the temperature field is faster, and better microstructure, plasticity, and toughness can be obtained in the welded joint.

### 5.2. Results of Stress Field 

**Figure 11:**
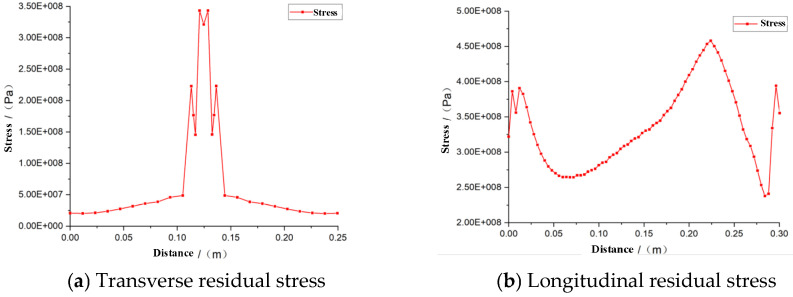
Variation in the residual stress of the weld cooling.

According to the optimization scheme of welding, the residual stress variation is shown in Figure 11.

When analyzing the welding residual stress in large thick-plate welded steel members, it is observed that the distribution range in the thickness direction is small. The stress is biaxial, and exhibits a plane state, consisting mainly of transverse residual stress and longitudinal residual stress. As shown in Figure 11, the welding residual stress is primarily concentrated in the weld zone. In the transverse residual stress variation curve, the value increases as it approaches the weld, reaching its maximum peak at the middle position, with a maximum residual stress of 345 MPa. At the same time, when far away from the weld area, the transverse residual stress changes from tensile stress to compressive stress, finally decreasing, gradually. In the longitudinal residual stress, it can be seen that the welding starting end is under pressure, the welding seam area is experiencing tension, and the overall distribution is relatively standard, which meets the welding requirements.

### 5.3. Results of Strain Field 

During the welding process, the deformation caused by welding will cause a huge waste of materials and workers’ labor, which will reduce the service life, and seriously affect the accuracy of the welded steel members, especially large thick steel members. Therefore, in the welding process, it is necessary to control deformation. According to the simulation results, the lateral and longitudinal displacement strains are shown in Figure 11.

**Figure 12:**
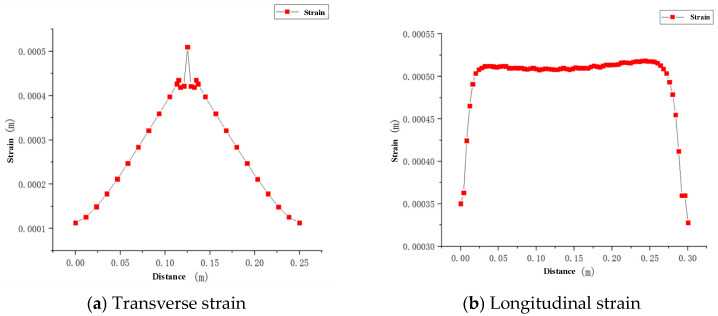
Variation in the welding–cooling residual strain.

From Figure 12, it is clear that, with the operation of the welding heat source, the strain increases gradually from the edge to the weld area; reaches its maximum at the center of the weld, with the maximum strain being 0.52 mm; then decreases gradually away from the weld. It can be seen that the longitudinal strain is very small at the beginning, and then rises sharply, which means that the temperature difference has not caused excessive strain at the beginning of the heat source loading. However, with the cooling, the strain increases gradually in the weld zone, due to the influence of residual stress. The deformation in the whole welded plate is small and evenly distributed, which meets the welding requirements.

### 5.4. Welding Test Detection

**Figure 13:**
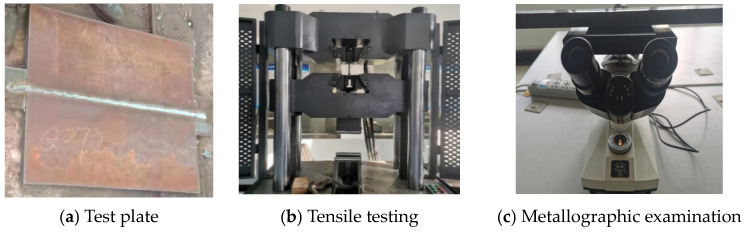
Experiment of the welded joints.

According to the welding optimization scheme, a test is carried out on the welded plate. After the welding is finished, and the welded part is cooled down, the mechanical inspection and macroscopic inspection are carried out on the welded plate, checking whether it meets the welding quality. The experimental welding plates and equipment are shown in Figure 13.

After the welding and cooling, the flat joint plate is tested, and the mechanical properties of its welding area are tested. The results are as follows.

1. The average tensile strength and yield strength of the weld zone are 565.5 MPa and 404 MPa, respectively, meaning that the test results show that the tensile properties of the welded joints are excellent, and meet the welding standards.
2. There is no crack in the welded joint, and it has an excellent plastic toughness, which meets the toughness requirements, and there are no pores, residues, cracks, or pits at the welded joints.
3. The highest hardness of HAZ is 194.8 HV; the average hardness is 188.525 HV. The hardness is lower than HV380, and meets the welding standard.
4. The welded plate has no significant deformation or distortion; the maximum deformation is 0.6 mm.

## 6. Conclusions

Taking Q345Qd bridge steel, through thermal–elastic–plastic finite simulation, the process parameters of the GMAW welding current, welding voltage, and welding speed are investigated, and the conclusions are as follows.

1. Based on the welding quality requirements of Q345qD for thick-plate bridge steel, the technical route of the GMAW welding process is established, and its parameters are studied. According to thermal–elastic–plastic analysis, the optimal welding parameters are utilized.
2. As results show that, with the gradual increase in the welding current, the maximum welding temperature can reach 3706 °C, the optimal welding current is 230 A, the optimal welding voltage is 32 V, and the optimal welding speed is 0.003 m/s.
3. According to the simulation analysis of single welding process parameters, the welding residual stress is mainly distributed in the weld zone; its value increases closer to the weld, reaching the maximum peak value in the middle position. The maximum residual stress is 345 MPa.
4. According to the experimental results, the average tensile strength and yield strength in the weld zone are 565.5 MPa and 404 MPa, indicating that the welded joint has excellent tensile properties.

The optimal parameters of GMAW welding will be applied to the welding robots, which will benefit greatly when it comes to improving the performance of the bridge.

## Figures and Tables

**Table 1:** Welding parameter setting.

Number of Beads	Welding Process	Protective Gas Flow Rate (l/min)	Electric Current (A)	Voltage (V)	Welding Speed (m/s)
1~2	GMAW	10~15	210~250	28~36	0.0026~0.0043
3~4	SAW	--	560~660	33~37	0.003~0.0046

**Table 2:** Parameters of the double-ellipsoid heat source model.

Sequence of Weld Paths	$a_{r}$ (m)	$a_{f}$	*b* (m)	*c* (m)
1	0.012	0.0036	0.006	0.007
2	0.006	0.0018	0.003	0.0045
3	0.011	0.003	0.005	0.003
4	0.016	0.0048	0.008	0.004

**Table 3:** Chemical composition of Q345qD.

	C	M_n_	S_i_	V	N_b_	T_i_	C_r_	N_i_
Standard	≤0.18	≤1.6	≤0.55	0.010~0.080	0.005~0.060	0.006~0.030	≤0.30	≤0.30
Certificate	0.16	1.48	0.21	0.010	0.0055	0.0067	0.20	0.10

**Table 4:** Setting of the welding process parameters.

Number of Plies	Welding Process	Protective Gas-Flow Rate (l/min)	Electric Current (A)	Voltage (V)	Welding Speed (m/s)
1	GMAW	10	230	32	0.003
2	GMAW	10	230	32	0.003
3	SAW	--	600	35	0.004
4	SAW	--	600	35	0.004

## Data Availability

Not applicable.
